# Breast Milk and COVID-19: From Conventional Data to “Omics” Technologies to Investigate Changes Occurring in SARS-CoV-2 Positive Mothers

**DOI:** 10.3390/ijerph18115668

**Published:** 2021-05-25

**Authors:** Flaminia Bardanzellu, Melania Puddu, Vassilios Fanos

**Affiliations:** Neonatal Intensive Care Unit, Department of Surgical Sciences, AOU and University of Cagliari, SS 554 km 4500, 09042 Monserrato, Italy; Puddu.melania@gmail.com (M.P.); vafanos@tin.it (V.F.)

**Keywords:** COVID-19, SARS-COV-2, antibodies, innate immunity, metabolomics, microbiomics, breast milk, breastfeeding

## Abstract

In this context of COVID-19 pandemic, great interest has been aroused by the potential maternal transmission of SARS-CoV-2 by transplacental route, during delivery, and, subsequently, through breastfeeding. Some open questions still remain, especially regarding the possibility of finding viable SARS-CoV-2 in breast milk (BM), although this is not considered a worrying route of transmission. However, in BM, it was pointed out the presence of antibodies against SARS-CoV-2 and other bioactive components that could protect the infant from infection. The aim of our narrative review is to report and discuss the available literature on the detection of anti-SARS-CoV-2 antibodies in BM of COVID-19 positive mothers, and we discussed the unique existing study investigating BM of SARS-CoV-2 positive mothers through metabolomics, and the evidence regarding microbiomics BM variation in COVID-19. Moreover, we tried to correlate metabolomics and microbiomics findings in BM of positive mothers with potential effects on breastfed infants metabolism and health. To our knowledge, this is the first review summarizing the current knowledge on SARS-CoV-2 effects on BM, resuming both “conventional data” (antibodies) and “omics technologies” (metabolomics and microbiomics).

## 1. Introduction

Mother to child SARS-CoV-2 transmission is still an open question and a topic of great relevance in such a pandemic period [1,2,3].

Transplacental transmission of SARS-CoV-2 and specific antibodies, previously considered doubtful [4], has been demonstrated, albeit in a few cases [5,6,7,8,9].

Dong et al., although did not detect SARS-CoV-2 in breast milk (BM) from a positive mother and in her newborn swab, found specific immunoglobulin A (IgA) and immunoglobulin G (IgG) in milk samples and serum IgG in the newborn since a month and half after birth, suggesting transplacental antibodies’ transmission [10].

The potential mother to infant SARS-CoV-2 transmission during breastfeeding arouses great interest among the scientific community, especially considering the benefits of breastfeeding and the disadvantages potentially related to its suspension in the offspring of affected mothers [11,12].

Some studies suggest the potential transmission of SARS-CoV-2 through BM of positive mothers [2,13,14,15], but such topic is still field of controversies [3,11,16,17,18,19,20].

In fact, a systematic review of 14 studies, dating back to April 2020, reported that 47 out of 48 samples collected from affected mothers and tested for SARS-CoV-2 were negative [21].

Subsequently, some studies showed SARS-CoV-2 in BM in few cases [2,22,23,24], although this is not currently considered the principal source of viral transmission [25].

Moreover, finding SARS-CoV-2 RNA in BM does not necessarily mean a viable potentially transmissible virus, as recent works have shown [2,22,23,26].

It should also be considered that BM of SARS-CoV-2 affected mothers can transfer to the newborn protective maternal antibodies against the virus [25]. IgA to SARS-CoV-2 excreted in BM of infected mothers could protect breastfed neonates, reducing viral transmission and disease’s severity [25].

As is widely known, most of BM-related benefits, in terms of antiviral properties and immunity, is conferred by the transmission of maternal antibodies [11], bioactive factors including lactoferrin [12] and a specific protective lactobiome [20].

Already from the colostrum, the newborn receives bioactive factors, including IgA, mucins, tryptophan, lactoferrin, α-lactalbumin, and growth factors, providing immunomodulation, anti-infective protection, and promoting gut mucosal development [24].

To date, little is known on BM innate immunity-related antivirals’ effects against SARS-CoV-2. Among them, lactoferrin seems potentially effective, based on its well demonstrated effects on other viruses [27] and on a single trial involving COVID-19 patients [28]; however, considering that a control group is absent in this prospective observational study, it should be further investigated if lactoferrin could potentially avoid the onset of a severe and prolonged form of COVID-19.

Due to the potential effects of SARS-CoV-2 on intestinal mucosa and on the respiratory system, most authors currently recommend breastfeeding even in mothers positive for the virus, as interrupting it would be more dangerous [1,3,24,29].

Moreover, it should be fully clarified if neonates of affected mothers should be breastfed directly or through expressed milk [30,31,32,33], and the use of donor human milk could be also considered [12].

Even the effects and safety of the current strategies for BM treatment in milk banks are under evaluation; i.e., a study from Walker et al. investigated the stability of SARS-CoV-2 inoculated in BM samples from healthy donors, to attest the efficacy of viral inactivation. According to this study, Holder pasteurization (63 °C for 30 min) and heating (56 °C for 30 min) completely inactivated SARS-CoV-2, differently from cold storage (4 °C or −30 °C). The same conclusion was reported by Unger et al. [34]. Thus, Holder pasteurization, currently used in milk banks, seems enough to inactivate SARS-CoV-2 viral load [34,35] without reducing SARS-CoV-2 antibodies, although neutralizing capacity could be lowered by such technique [36].

The purpose of this narrative review is to summarize the current knowledge regarding the presence of anti-SARS-CoV-2 antibodies in the milk of affected/positive mothers, and the studies that had applied metabolomics to the milk analysis of COVID-19 positive mothers, trying to interpret metabolic changes in relation to the infection and the specific lactobiome related to the SARS-CoV-2 pandemic. Moreover, the potential link between BM lactobiome of SARS-CoV-2 positive mothers and the future health of the offspring was investigated. We discussed available literature, found on MEDLINE, and updated 12 March 2021, using COVID-19, SARS-COV-2, antibodies, metabolomics, microbiomics, breast milk, and breastfeeding as key words.

To the best of our knowledge, this is the first review summarizing the current knowledge on SARS-CoV-2 influence and effects on BM, resuming both “conventional data” (antibodies) and “omics technologies” (metabolomics and microbiomics).

## 2. Breast Milk, SARS-CoV-2 and Antibodies

Although previous SARS-CoV-2 findings in BM [2], from the results of Pace et al., viral transmission would not seem possible during breastfeeding; on the contrary, infected mothers’ BM could give the tools to fight or attenuate neonatal infection, through IgG or IgA specific antibodies [11] or other not yet fully clarified factors.

To date, some studies investigated the presence of SARS-CoV-2 antibodies in human BM [10,11,37].

In the study of Pace et al., RT-qPCR did not detect SARS-CoV-2 RNA in milk samples collected from 18 COVID-19 affected mothers (although none of them was hospitalized for a severe symptomatology), despite a very small evidence of viral RNA in breast skin swabs; thus, they suggest that such a “cutaneous” transmission should be also investigated (maybe depending on contamination by hands, droplets or other sources) [11].

In most of these milk samples, SARS-CoV-2-specific antibodies were present (76% IgA and 80% IgG), with higher concentrations of the first class and, consequently, BM produced by mothers with COVID-19 acquires the in vitro ability of viral neutralization (62% of cases) [11].

Specific IgA to SARS-CoV-2 were also found in 97% of samples (study on 39 women) in the study of Demers-Mathieu et al. [38] and were positive at 3 and 6 days after delivery [25] and up to 6.5 months after infection in a positive mother [36] in other studies.

According to these recent observations, breastfeeding should be continued in case of mild-to-moderate maternal COVID-19 [11].

On the surface of coronaviruses, the spike protein (S) is composed by two specific subunits (S1 and S2), determining viral specificity and infectivity; in detail, S1contains the receptor-binding domain (RBD), involved in cell surface recognition, while S2 is mostly involved in the fusion [39].

According to the results of Fox and co-workers, in all BM samples (*n* = 15) collected from COVID-19 positive mothers, IgA against to the full spike protein of the SARS-CoV-2 were present, instead of prepandemic collected samples, while 12 out of 15 samples also contained IgA against the receptor binding domain (RBD) of the SARS-CoV-2 spike protein, mostly being secretory IgA (sIGgA); 67% of samples also showed IgG or IgM anti-RBD [37].

In COVID-19 affected mothers’ samples of BM, anti-RBD IgA and IgG, anti-spike S2 and antinucleocapsid (N) IgG, and IgG anti-HCoVOC43 were detected, higher than pre-pandemic samples [11].

Moreover, in vitro, 62% of samples collected from COVID-19 mothers neutralized SARS-CoV-2 infectivity, differently from all the prepandemic collected samples; such ability was mostly but not only correlated with RBD-reactive IgA or IgG, since other not clarified factors could be involved [11].

Demers-Mathieu et al. measured the levels of SIgM/IgM, IgG and SIgA/IgA reactive to SARS-CoV-2 S1 and S2 subunits (S1 + S2) and nucleocapsid protein in BM samples collected during COVID-19 pandemic and compared them with prepandemic controls. Moreover, antibodies between vaccinated and unvaccinated women were measured, and antibodies between symptomatic and healthy mothers (during pandemic) were also investigated [38].

Samples collected during pandemic from symptomatic mothers showed higher S1 + S2-IgG levels than asymptomatic ones, while S1 + S2- and nucleocapsid-reactive IgG were higher in the pandemic group than prepandemic samples [38].

BM antibodies, protecting the newborn against SARS-CoV-2 infection, could derive from maternal infection, even if asymptomatic, or even by previous infections by other viruses [38].

In fact, some antibodies, i.e., to several human coronaviruses (HCoVs) including SARS-CoV-1, could cross-react to SARS-CoV-2, resulting protective, and the purification of these antibodies could be employed as therapeutic strategy, following confirms in vivo and in vitro [11,38,39].

The group of Demers-Mathieu demonstrated the presence of some cross-reactive antibodies among SARS-CoV-2 and common HCoVs, such as S1 and S2 subunits of HCoV-OC43 and HCoV-229E, by comparing BM samples from three groups of women: COVID-19 PCR positive mothers, mothers presenting viral symptoms, and a prepandemic group of mothers (control group). As result, SARS-CoV-2 IgG to S2 subunit was higher in the first two groups [39].

The cross-reactivity between SIgA and SIgM in the groups of COVID-19 PCR group and the control group could be related to the polyreactivity to the subunits S1 and S2; the neutralizing capacity in the three groups requires further clarification too.

Antibodies S1+S2 reactive HCoV-OC43 IgG were higher in COVID-19 cases than in controls, while HCoV-229E IgG were comparable, suggesting that cross-reactivity is higher between the S2 subunits of SARS-CoV-2 and β-coronaviruses than α-coronaviruses, and could result protective [39].

Potential cross-reactivity among SARS-CoV-2 antibodies and non SARS common cold coronaviruses is also under evaluation [40].

Root and colleagues also hypothesized that vaccination against *H. Influenza* type B and *S. pneumoniae,* mitigating influenza-related complications, could also reduce COVID-19 morbidity and mortality and therefore, following adequate demonstrations, could represent strategies to face the current pandemic [41].

## 3. Breast Milk, SARS-CoV-2 and Metabolomics

Although SARS-CoV-2 transmission through BM of COVID-19 affected women is still under study, it could affect its composition, being BM an expression of continuity between the mother and her infant.

The metabolomics study of BM metabolites (molecules weighing <1500 daltons) could help to understand the possible impact of viruses on such biofluid, providing a comprehensive and dynamic analysis of BM. The several thousands of molecules that make up the human metabolome are produced by the genome of the host and its microflora, or may be derived from exogenous factors such as drugs [42].

Therefore the study of BM metabolome could highlight potential metabolites representing specific biomarker for COVID-19 reflecting metabolic alterations in the host induced by the infection.

To date, a single study investigated BM metabolome in case of maternal SARS-CoV-2 infection; Zhao et al., examined colostrum samples from four SARS-CoV-2 positive and two negative mothers whose infants were negative [43]. Positive mothers developed mild symptoms characterized by fever (<38 degrees °C) and, in only one case, a dry cough. Lipidomics, proteomics and metabolomics analysis were performed on all six BM samples, which were negative for serological tests and viral SARS-CoV-2 RNA. Data obtained were subjected to pathway analysis and, finally, were included in a regulatory network.

Lipidomics provided unremarkable data; in fact, no separation occurred between the two classes with Principal Component Analysis (PCA) and only 13 metabolites were different, out of 504, in samples from positive mothers.

Proteomics highlighted 88 different expressed proteins (DEPs), mostly involved in inflammatory processes and immune response. In particular, the pathway analysis showed a down-regulation of markers of neutrophil degranulation, including CD44 and complement factor propendin, of platelet degranulation and leukocyte migration, of granulocyte neutrophil survival and homeostasis (PRKAR2A), and even lymphoangiogenesis (ADAMTS3) [43].

Although conducted on a small number of samples, the identified DEPTs correlate with processes altered in serum samples collected from COVID-19 adults patients too, in the only two studies that, to the best of our knowledge, employed proteomics together with metabolomics [44,45].

Shen et al., on a cohort of 118 individuals (65 severe and 65 non-severe COVID-19 patients versus 53 non-COVID controls) identified 105 proteins differently expressed in sera from COVID-19 vs. non-COVID patients; among them, 50 proteins are involved in 3 main pathways: complement activation (down-regulation of properdin and 2 apolipoproteins), platelet degranulation (down-regulation of platelet expressing chemokines), and macrophage function (down-regulation of several apolipoproteins). In addition, in the comparison between severe and non-severe COVID-19 patients, some proteins, representing biomarkers of the acute inflammatory phase of viral infections, were significantly increased in the first group. These included serum amyloid 1, 3, 4, SERPIN 3, and, as expected, C-reactive protein (CRP) [44].

Su and colleagues performed a multi-omic study in which disease severity, common laboratory tests, transcriptomics of cells involved in immune processes, proteomics, and serum metabolomics were combined through sophisticated statistical methods. Among the very large number of samples under study, significant differences emerged by comparing healthy controls versus mildly symptomatic patients and also mild versus moderate COVID-19 patients, while moderate and severe patients almost overlap. These differences concern the up-regulation of proteins involved in inflammatory processes such as IL-6, IL-10, CCL7 (chemokine ligand 7), and KRT 19 (Keratin-19), necessary for the organization of muscle fibers and marker of tissue damage [45].

In the study by Zhao et al., the only metabolomics analysis on BM, six BM samples were analyzed by Liquid Chromatography-tandem mass spectrometry (LC-MS/MS). PCA clearly separated samples from COVID-19 mothers and controls. The analysis of the 79 most significant metabolites highlighted pathways of protein degradation, aminoacyl-tRNA biosynthesis and aromatic amino acid metabolism [43].

Seventeen metabolites (aromatic amino acids and their derivatives) differentiated samples collected from the two groups of mothers. Tryptophan, phenylalanine, and tyrosine and their catabolites were significantly reduced in samples from COVID-19 affected mothers. The inclusion of these amino acid metabolites in a regulatory network with DEPTs revealed that 10 out of them, including phenylalanine and its catabolite phenethylamine, and indol-acetic acid, are interconnected with DEPTs involved in immune processes.

Since the composition of BM generally mirrors that of maternal blood, in the present work we aim to compare the results of the study by Zhao et al. with metabolomics studies performed on the serum of adults with COVID-19, always taking into account the limitations of the study, due to the small number of BM samples examined [43].

### 3.1. SARS-CoV-2 and Tryptophan Metabolism

As aforementioned, the tryptophan pathway is one of the most affected in the study by Zhao et al. [43]. Metabolomics studies conducted in adults also found that, among aromatic amino acids (AAAs), tryptophan undergoes the greatest changes, and its reduction in COVID-19 patients compared to healthy subjects [44,46,47,48,49,50] agrees with the results on colostrum [43].

However, there is some discordance regarding the stage of the disease in which tryptophan was reduced. In the study of Thomas et al., the reduction is more pronounced in case of more severe disease [47]; in the study of Barberis et al. it decreases from the earliest stages of disease but rapidly drops in patients admitted in ICU (intensive care units) [46]. Shen et al. found low levels of tryptophan in both in severe and non-severe patients [44], while Su et al. only detected its reduction in mild patients [45].

In colostrum, kynurenine was decreased in samples from COVID-19 mothers [43], while in several studies on adults [44,45,47,51] the kynurenine pathway was one of the most affected and kynurenine and/or some of its metabolites were significantly increased in COVID-19 patients. In the study of Thomas et al. kynurenine positively correlated with disease severity (assessed by interleukin 6-IL6 concentrations) and its metabolic derivatives, kynurenic acid, picolinic acid, and nicotinic acid were also increased [47]. Shen et al. reported similar results, with a significant increase occurring only in severe patients. In their study, kynurenine was one of the most discriminating metabolites between severe-COVID-19 patients and the others [44].

Furthermore, in the study of Su and colleagues kynurenine was increased in COVID-19 patients, but only by comparing mildly symptomatic ones versus healthy controls. Kynurenine was one of the five metabolites mostly correlated with the percentage of a novel immune cell subset exhibiting transcripts with high cytotoxicity, emerging in moderate cases, and increasing with disease severity. It was also significantly correlated with the percentage of immune cell subpopulations associated with disease severity (Proliferative exhausted CD8+ T cells, Cytotoxic CD4+ T cells, Proliferative exhausted CD4+ T cells, Proliferative NK cells, Pro-inflammatory and antigen presentation deficient monocytes, Plasma cells) [45].

Indol-acetic acid, a gut flora-derived metabolite produced from tryptophan, and nicotinamide, a final metabolite of the kynurenine pathway that is part of the nicotinamide adenine dinucleotide (NAD), were among the top 15 metabolites differentiating COVID-19 versus non-COVID-19 subjects in the study of Blasco et al., in which the nicotinic acid-nicotinamide pathway was the most affected [49].

In the study of Fraser et al., kynurenine was the first of the eight most relevant metabolites, showing a fivefold increase in COVID-19 patients versus healthy controls. The second metabolite, arginine, was significantly reduced. The kynurenine/arginine ratio provided a 98% classification accuracy (*p* = 0.005) between severe and non-severe COVID-19. The reduction in arginine may indicate a consumption following its enhanced requirement during the acute phase of the disease, being a nitric oxide precursor and tissue repairer [51].

In the study of Song et al., several amino acids, including tryptophan, were reduced in mild and moderate patients. In addition, 5H-tryptophan (5-HT) was increased and represented one of the top metabolites discriminating COVID-19 patients from healthy ones [50].

In the study of Cai et al. the activation of the kynurenine pathway was correlated with the sex of the patients (as known, males are more affected by COVID-19). Specifically, kynurenic acid and kynurenic acid/kynurenina ratio were strongly associated with the disease severity and inflammatory cytokine levels in males. In females, the correlation with lower cytokine numbers and T cell activation occurred, but not with disease severity [48].

Tryptophan is a precursor of several biologically active metabolites through three metabolic pathways. In the first pathway, a small percentage of tryptophan is hydroxylated to 5-HT, to produce serotonin and melatonin. The second pathway leads to the production of kynurenine through the enzyme indoleamine 2,3-dioxygenase-1 (IDO1), expressed in peripheral lymphatic organs (including Peyer’s plaques), and in the lung. Kynurenine can be converted to kynurenic acid or ultimately results in the cellular cofactor nicotinamide adenine nucleotide (NAD), an important oxidizing agent in cellular respiration. The third pathway implies the direct action of intestinal flora on the tryptophan molecule, with the production of indole derivatives (through tryptophanase), and tryptamine (through tryptophan-decarboxylase). The end products of the kynurenine pathway, particularly kynurenic acid, appear to be ligands for the aryl hydrocarbon receptor (AhR) considered a key component of the immune response in many cell types, such as intraepithelial lymphocytes, Th17 cells, innate lymphoid cells, macrophages, dendritic cells (DCs), and neutrophils [52].

From the above considerations we can assume that tryptophan reduction, found in metabolomics aforementioned studies, could be due to an increased consumption; this could result from the demand for NAD in COVID-19 with greater respiratory impairment, the increase in its catabolism by the direct action of the intestinal flora resulting in abundance of indole derivatives (with anti-inflammatory and immunoregulatory effect) and ahR ligands (able to modulate locally or remotely immune homeostasis and barrier physiology). Finally, the increased tryptophan consumption could derive from the activation of IDO, with consequent increase of kynurenine derivatives involved in several biological processes such as neurotransmission, inflammation and immune response.

IDO antiviral action of has been proved in several studies and for different viruses such as measles, herpes, influenza, parainfluenza, respiratory syncytial virus. Specifically, parainfluenza virus type 3 (HPIV3) is strongly inhibited by this enzyme in the lung epithelium [53,54,55,56,57].

Since COVID-19 predominantly respiratory component, being IDO expressed in the lung where ahRs are also present in both antigen presenting and epithelial cells, its activation in the tryptophan pathway could promote recovery or prevent its evolution to more severe respiratory forms.

On the contrary, the early gut involvement, often occurring in children or adults, could damage microflora and consequently reduce ahR ligands, impairing prognosis.

In conclusion, and always considering that the study of Zhao et al. was performed only on four colostrum samples that came from mothers with mild symptoms [43], we can argue that the results concerning tryptophan metabolism, largely contrast with those carried out on the blood of adults with COVID-19, even considering only mild patients.

It would seem that colostrum is oriented in a pro-inflammatory and not anti-inflammatory way and this finding is in agreement with the interconnection of most of the top metabolites of colostrum with the DEPTs involved in inflammatory and immune processes. Moreover, being tryptamine a ligand for serotonin 5-HT4 intestinal receptors, to which is attributed a role in the regulation of intestinal peristalsis and motility, its reduction, as expression of intestinal flora damage, may affect these functions in the infant.

### 3.2. SARS-CoV-2, Phenylalanine and Tyrosine Metabolism

Several metabolomics studies in COVID-19 adult patients show the involvement of the tyrosine and phenylalanine pathways (along with that of tryptophan), as in the study on BM [43]. In the study of Barberis et al., tyrosine and phenylalanine biosynthesis, aminoacyl-t RNA degradation, and phenylalanine metabolism seem involved in COVID-19. Blood levels of phenylalanine and tyrosine increase in non-critical patients and then dramatically decrease in ICU patients. Other amino acids, such as glutamine, histidine, proline, and valine, show a similar trend [46].

Thomas et al., who classifies the disease by serum IL6 levels, found that the tyrosine pathway is one of the most affected. Increased phenylalanine is found only in patients with higher IL6 levels and, as in the previous report, there is reduction of several amino acids (alanine, glycine, serine, glutamine, histidine cysteine, and taurine) [47].

In a large case series on patients in the acute phase of the disease, Bruzzone et al. found a consistent increase in phenylalanine (81%) and a 4% reduction in tyrosine [58].

In the study of Su et al., phenylalanine increased in mildly symptomatic patients compared with the healthy ones and also in mild cases compared with severe ones, whereas it was reduced in severe patients compared with the healthy controls. Phenylalanine, along with kynurenine, was one of the five metabolites whose increase was correlated with the percentage of the immune cell subpopulations associated with disease severity and listed above. In the same study, tyrosine was decreased in mild patients compared with healthy controls [45].

Pàez-Franco et al., in a preprint study comparing healthy individuals with mild and severe patients, found that phenylalanine is one of the 15 metabolites that differentiate the healthy from mild and severe patients, while it is not among the discriminating when comparing mild to severe cases. Moreover, in this study, several amino acids decrease in the mild patients compared to the controls (methionine, glutamine, threonine, isoleucine, and leucine) [59].

Phenylalanine was one of the 11 top metabolites in the study of Meoni et al. It increased in COVID-19 patients, in contrast to histidine, glutamine, glycine, and alanine that were reduced. Tyrosine was also reduced, although not significantly. Among the 30 COVID-19 patients, the disease evolved into ARDS in 19, but only marginal metabolomics differences were present in these subjects compared with the less severe ones [60].

Taking into account the differences between the various studies (methodology, number of cases, and classification of patients), and analyzing the metabolites considered in the present work (those indicated by the study on colostrum of infected mothers), their results seem to highlight, in the early stages of COVID-19, an increased protein and energy demand, justified both by the viral need to replicate and by the host necessity to defend itself.

In agreement, several amino acids increasing at the beginning of the disease and/or in the mild–moderate patients are employed as energy source by entering the Krebs cycle or gluconeogenesis. The subsequent consumption leads to their decline in advanced forms of COVID-19. This is also the trend showed by phenylalanine, which increases in mild [45,46,59] and is reduced in severe cases [46] and by tyrosine [45,46,58].

The increased protein requirement in the early stages is also confirmed by the involvement of the aminoacyl-tRNA pathway [46], which is critical in protein synthesis and seems to have a role in regulating immunity against infection [61]. As already pointed out, it is difficult to draw conclusions about the significance of the changes in the abundance of selected metabolites in colostrum of COVID-19 affected mothers. The involvement of the aminoacyl-t-RNA pathway might indicate the activation of protein synthesis. As for phenylalanine, significantly reduced in colostrum, all its metabolic pathways are under-activated. In fact, both the production of tyrosine (through phenylalanine hydroxylase), and phenyl-ethylenediamine (through aromatic L-amino acid decarboxylase), and trans-cinnamic acid, by an intestinal flora lyase were reduced.

Moreover, in agreement with the adult studies in which phenylalanine was increased in mild patients, we would have expected it to have a similar behavior in colostrum, since the four mothers investigated had mild infection.

Metabolomics on the serum of the same mothers would have helped perhaps to understand and we hope that studies on larger samples, in patients with disease of different severity, on BM collected at different stages of lactation, with simultaneous analysis of blood metabolites of the mothers, will be performed in the future. Their results could help to understand the consequences of COVID-19 disease in breastfed infants and to direct possible behaviors in this regard.

## 4. Breast Milk, SARS-CoV-2, and Microbiomics

As reported above, many of the top metabolites potentially representing biomarkers for COVID-19, both in BM and serum of adult patients, derive from the gut flora. The greatest ambition of researchers in this field is to identify, through the study of the microbiome of the various biofluids, the microorganisms involved in COVID-19 and, through metabolomics, the metabolites produced or modified by them and their role in the clinical course and in the disease outcome.

While there are preliminary studies of the application of this flow-chart in the adult, BM microbiome has never been investigated in pregnant COVID-19 affected women. The analysis could lead to innovative results that would help to evaluate not only COVID-19 consequences on maternal microbiota, but also the influence of maternal infection on BM microbial populations, so important, as known, for the development of the immune system of the newborn and therefore influencing its future health. Long-term follow-up, although crucial to establish the future implications of COVID-19 on pregnancy and newborn, has not been performed given the “young age” of the virus. Currently, we can only examine the studies carried out so far in adults, assuming that their results can be at least partly similar to those eventually performed on pregnant women, and investigating whether these results might affect the composition of BM microflora.

Emergent set of data talks about the role of microbiome in predicting the severity of COVID-19 [62,63]. In our organism, gut microbiome can be considered the major regulator of immune response. Good bacteria play a protective action against infections, as well as a regulatory function of the metabolic and hormonal system [64].

The lack of good bacteria in the gut of patients with COVID-19 could affect the severity of the disease. Recent studies involving patients with different severity of disease seems to confirm this supposition [65,66].

A study on the fecal microbiota carried out with shotgun metagenomics in patients with COVID-19 during hospitalization, showed alterations significantly correlated with disease severity, first in a pilot study of 15 individuals [65] and later in a larger one involving 100 subjects [66] until discharge and swab negativity.

Symbiotic bacteria resulted significantly reduced, while there was an enrichment of opportunistic flora. In both studies, patients with COVID-19 did not significantly differ from healthy controls in fecal microbiome diversity, except when antibiotics were used.

In the first pilot study, different commensal genera of Firmicutes phyla (Eubacteriaceae, Ruminococcaceae, and Lachnospiraceae), were reduced in COVID-19 patients. Symbiont bacteria, *F. prausnitzii* spp. and *A. onderdonkii* spp. were the principal species showing a negative correlation with COVID-19 severity. Three bacterial members of the genus *Coprobacillus*, the species *C. ramosum* and *C. hathewayi*, were the top bacteria positively associated with COVID-19 disease severity.

Four Bacteroidetes spp. showed inverse correlation with fecal SARS-CoV-2 load while *Erysipelotrichaceae bacterium* 2_2_44A, a Firmicutes species, showed the strongest positive correlation with fecal SARS-CoV-2 load [65]. Erysipelotrichaceae is supposed to be involved in gut inflammation-related [65].

In the second study, Actinobacteria and Bacteroidetes were respectively decreased and increased. Some Firmicutes (*F. prausnitzii* spp., *E. rectal* spp.) were significantly reduced and others (some *Ruminococcus* spp.) increased in COVID-19 patients. There was also a reduction of Actinobacteria (i.e., *B. adolescentis* spp.) and increase in Bacteroidetes (*B. doreus* spp., *B. ovatum* spp.) and Verrucomicrobia phyla (*A. muciniphila* spp.). Most significant bacteria negatively correlated with disease severity were *F. prausnitzii* spp. and *B. bifidum* spp. Interestingly, dysbiosis also continued after clearance/recovery of SARS-CoV-2 infection [66].

Moreover the role of microbiota in modulating host immune response, and potentially influencing disease severity also in COVID-19, is confirmed by the inverse correlation of bacterial species depleted in COVID-19 patients, with plasma concentration of several cytokines (IL10, TNF-α, CXCL10, CCL2). These species include *B. adolescentis* spp., *E. rectal* spp., and *F. prausnitzii* spp. Conversely, *A. muciniphila* spp. and *B. dorei* spp., in the COVID-19 cohort, was positively correlated with IL-1β, IL-6, and chemochine IL8. The significance of these changes increased with the disease severity [66].

In conclusion, the results of the aforementioned studies [65,66] indicate that the immune response to COVID-19, and consequently the severity of the infection, could be influenced by the disruption of the bacterial flora that often affects these patients.

Another Chinese study of a group from Zhejiang University compared 30 COVID-19 patients with 24 affected by H1N1 and 30 healthy controls [67]. None of the subjects had received antibiotic therapy or probiotics for at least 4 weeks. Even in this case, the opportunistic bacterial flora was significantly increased at the expense of commensal flora. In both cases, COVID-19 and H1H1, there was a significant decrease in gut microbiota diversity and abundance. Firmicutes were increased in both, but especially in H1N1, while Actinobacteria were significantly increased in the former and reduced in the latter.

A reduction of butyrate-producing bacteria (Ruminococcaceae and Lachnospiraceae) occurred in both groups and a dramatic increase of Streptococcaceae only in COVID-19 subjects. At the genus level, the increased relative abundance of *Streptococcus* spp., *Rothia* spp., *Veillonella* spp., *E. bacterium* spp., and *Actinomyces* in COVID-19 patients was positively correlated with CRP and D-dimer levels, while the decrease abundance of *Agathobacter* spp., *Fusicatenibacter* spp., *Roseburia* spp. was negatively correlated mainly with CRP, plateletcrit (PCT), or D-dimer levels. In H1N1 but not in COVID-19 patients there was a positive correlation of species increased with inflammatory cytokines IL-2, IL-4, and IL-6.

Preliminary data on 990 subjects suggest that gut microbiota may predict the susceptibility of normal individuals to severe COVID-19 [68]. Starting from the data of the proteomics study of Shen et al. [44] on patients with COVID-19 disease of different severity (reported in the previous paragraph), the same group, through sophisticated machine learning methods, applied the proteomic risk score to a large population of healthy individuals. Integration with multi-omic data on proteome and inflammatory biomarkers resulted in a significant correlation of this score with CRP and TNFα, especially in the elderly people who, as known, represent the most susceptible population to severe forms of disease.

At the same time, the authors identified fecal microbial profiles that, still in healthy individuals, predicted the proteomic and cytokine biomarkers of COVID-19. A core microbiota of 20 OTUs belonging to the genus *Bacteroides, Streptococcus, Lactobacillus,* to the family of Ruminococcaceae, Lachnospiraceae, and to the order of Clostridiales significantly correlated with the proteomic score and, also in this case, especially in the elderly peolple. Eleven OTUs were associated with the cytokine profile. Among them, *Bacteroides, Streptococcus,* and *Clostridiales* were negatively correlated, while *Lactobacillus, Ruminococcaceae, Lachnospiraceae and Blautia*, positively.

Finally, through fecal metabolomics, they identified three main metabolic pathways that could potentially associate the gut microbiota with susceptibility to COVID-19. These pathways include the biosynthesis of aminoacyl-tRNA, arginine, branch chain amino acids [69].

In summary, the study highlights the ability of gut microbiota to identify healthy individuals susceptible to COVID-19.

However, why are we taking such an interest in the microbiota of adults with COVID-19?

As we specified above, the study of the microbiota of pregnant women would be of great concern because the intestine of the mother is supposed to contain most of the seeds from which microorganisms will develop in BM as well as BM contains the seeds to be sown in the intestine of the infant. In addition, maternal gut microbiota can modify that of BM through the bacteria metabolic products, with relevant implications.

Unbalance in microbiota, due to different reasons, determines dysbiosis, which could disrupt the aforementioned physiological seeding. There is now wide evidence that an altered early human gut microbiota compromises the achievement of a stable microbiota later in life and makes the individual more susceptible to various diseases long after birth [68,70,71,72].

This is what might happen when COVID-19 and associated dysbiosis affects pregnant women.

Table 1 summarizes the functions performed by gut commensals found altered in microbiome studies of patients with COVID-19. Furthermore, their correlations to several aspects of host physiology and disease are discussed.

### From “More Gut in the Lung” to “Less Gut in the Lung”?

Through non-cultural techniques (pyrosequency), the presence of gut-associated obligate anaerobic bacteria has been demonstrated in maternal feces, BM, and neonatal feces, which share *Bifidobacterium, Bacteroides, Parabacteroides* genera, and members of the Clostridia order (*Blautia* spp., *Clostridium* spp., *Collinsella* spp., and *Veillonella* spp.). Several butyrate-producing members of Clostridia order (*Coprococcus* spp., *Faecalibacterium* spp., and *Roseburia* spp.) were also shared between BM and neonatal gut [89,90].

Nonetheless, pyrosequencing does not clarify whether the bacteria found in the different biofluids are alive or dead; however, in other reports, *Bifidobacteria* spp. and *Lactobacillus* spp. have been identified at the three sites by cultural techniques, proving that at least some bacteria, including obligate anaerobes, can be vertically transmitted from mother to newborn [90,91,92]. Gut bacteria can move to the mesenteric circle through the entero–mammary pathway, reaching mammary gland and therefore being transferred to neonatal gut through BM. This represents one of bacterial routes of transmission (together with skin and mouth) [93,94,95].

A recent interesting study has shown that the number of bacteria transmitted from the mother to the newborn is significantly lower if BM is pumped and the neonate is fed from the bottle [96,97].

Bacterial translocation from the gut to extra intestinal sites, previously considered possible only in case of diseases (cancer, autoimmune diseases, etc.) [98] seems to occur even in healthy individuals, although in small amounts, and involve various tissues, including the mammary gland. In experimental animal models, bacterial cells have been found in gut-associated lymphoid (GALT) tissue already in late pregnancy [93,94]. This phenomenon could educate the newborn immune system to be tolerant towards maternal bacterial species and alert him in case of dangerous changes in the local lymph nodes.

The use of germ-free (GF) animals suggests that interactions with specific communities of microbes directly modulate GALT development, whose structure and function are deeply perturbed in the absence of gut commensals. Many of the alterations found in GF animals are reversible after the re-establishment of the normal microbiota; however, if colonization does not occur in a specific time-window, complete maturation of the intestinal immune system is never achieved [99]. In the rodent, this time-window correlates with the suckling period. In humans, the colonization is supposed to respect a temporal window too, to be effective in determining a positive imprinting on the immune system; otherwise, the shift from a window of opportunity to one of vulnerability resulting in susceptibility to diseases such as asthma, allergy and inflammatory bowel disease. Examples include infants born by cesarean section or those treated with antibiotics in the first few months of life or whose mothers were treated with antibiotics during delivery or at the end of pregnancy, which various studies have indicated as susceptible to these diseases as a result of disruption of the intestinal microbial balance [99,100,101].

Many of the bacteria identified with pyrosequency in maternal feces, in BM, and in newborn feces correspond to commensals found altered or correlated with severity of disease and its biomarkers in studies investigating microbiota in COVID-19 patients [66,67,69,102]. We speculate that the dysbiosis in pregnant women affected by COVID-19 could compromise the translocation of good bacteria from the mother’s gut to BM, impairing the immune system development of the infant. Much has been said about the impact of an altered translocation of bacteria from the gut to the lung in cases of severe dysbiosis, of that “more gut in the lung” able to worsen the respiratory course of a viral disease including SARS-CoV-2 [62,103,104,105,106]. In our topic, the “less gut in the mammary gland” is likely to have more relevance than the “more gut”, although this can be also present and ready to fill the niches of the “less gut”.

If these speculations were confirmed, new preventive and therapeutic perspectives would open up. Research has made great progress in the study of probiotics and their use may represent an interesting perspective [62]. After an initial optimistic period in which it seemed that COVID-19 was less severe in children, currently, also because of the new variants, the number of hospitalized children in a serious condition is increasing. Even if most of them recover, the literature points out the need of a long distance follow up after discharge. If this policy were implemented, we would be able to understand if COVID-19 related maternal dysbiosis may also compromise neonatal long-term fate.

## 5. Conclusions

The current evidence seems to sustain the beneficial effects of BM also in the case of mothers affected by SARS-COV-2, who should continue to breastfeed [24,107,108].

The analyzed studies support the possible protective effect of BM against COVID-19 also for the transfer of antibodies that can exert an antiviral action protecting respiratory and gastrointestinal systems [10,37].

Therapeutic perspectives have been proposed by the use of specific antibodies from BM, since specific IgA and other classes of antibodies seems to persist during lactation in affected/immunized mothers for at least seven months [109]. In fact, antibodies could be potentially collected, purified and used to prevent SARS-CoV-2 infection or to reduce symptoms in infected subjects [24], although specific data on anti-SARS-CoV-2 titers duration are not available.

Antiviral specific treatments would be highly required to face the COVID-19 pandemic. The role of antimicrobial peptides (AMPs), biomolecules belonging to adaptive immunity, including lactoferrin, could be promising preventing or mitigating SARS-CoV-2 infection [12,27,110,111,112].

The potential link between COVID-19 and gut dysbiosis is matter of great interest [63], and intestinal bacteria were also found in the lungs of affected patients [38,113]; therefore, BM could reduce COVID-19 severity acting on intestinal gut [38], modulating immune response against SARS-CoV-2 [24].

Much progress has been made in the field of COVID-19, but many issues remain unclear. Future research applied to BM will help us understand the most suitable therapeutic interventions and the potential link between metabolome–lactobiome and future infant health.

## Figures and Tables

**Table 1:** Gut commensals involved in COVID-19 and their correlations to host physiology and diseases.

	Bacterial Species	Study Findings
Bifidobacterium	The most abundant species in early childhood. Progressive decrease with age (5–10% in adults). Increasing of their abundance before weaning in BF infants promoting by HMOs [68]. Producers of SCFAs (acetate) and lactate through their saccharolytic activity, providing specific immune stimulation and acidification (protective against infections) of the intestinal environment. Cross feeders for butyrate producing bacteria. Promotors of bacteria–bacteria talk important for intestinal homeostasis and future gut microbiota [68,73]. Interaction with the human intestine favored by their extracellular structures ✓ The exopolysaccharide protects them against the recognition by immune system [68]. It reduces apoptotic signaling in gut epithelial cells mitigating the pro-inflammatory response in intestinal bowel diseases [68,74]; ✓ Fimbriae ensure the adhesion to intestinal cells, preserving the permeability of the intestinal mucosa promoting interaction with the host immune system and microbe-microbe interaction [68]. ✓ Serpine is a protease inhibitor with protective action against host proteases [68]. Promote IL10 production by dendritic cells [75].	Reduced in fecal microbiota of COVID-19 patients [66,67]. Negatively correlated with IL10* and INFα in hospitalized patients [66]
Lactobacillus	<1% of the whole intestinal microbiota and only 0.3% in the colon. Higher in breastfed infants up to 6 months. Protective role against infections due to the production of lactic acid (< intestinal pH), and acetate by the degradation of host-carbohydrates [68]. Involvement in tryptophan metabolism resulting in production of indole catabolites, interacting with the intestinal ahRs which promote intestinal barrier and protect from opportunistic pathogen [76].	Positively correlated with proteomic risk score for COVID-19 and with IL6 and IL10* [69].
Fecalibacterium prausnitzii	Considered the “gatekeeper” of intestinal health it is one of the most abundant and relevant commensal bacteria of the human gut. Immuno-modulatory properties and anti-inflammatory effects in murine models of colitis. Acetate consumer and butyrate-producer. Inducing IL-10 in vitro. Able to decrease IL8 concentration.Reduced in metabolic and gut diseases as Crohn’s disease, celiac disease, colorectal cancer, obesity as well as in elderly people [68,77].	Significantly reduced in hospitalized COVID-19 patients. Negatively correlated with the disease severity [65,66] and to the levels of IL10* and TNFα [66].
A. muciniphila	Considered the “sentinel of the gut” It colonizes the intestinal tract during infancy and reaches 1–4% of the fecal microbiota in adulthood. Cross feeders for butyrate producing bacteria by mucus degradation, allowing their anti-inflammatory properties. Reduced in metabolic inflammatory diseases such as diabetes, obesity, and IBD in mice and humans [78,79,80]. Involvement in tryptophan metabolism resulting in production of indole catabolites [81].	Increased in hospitalized COVID-19 patients and positively correlated with some cytokines [66]. Compensatory effect? Can the increase allow the growth of good butyrate-producing bacteria, counteracting the inflammation?
Ruminococcaceae and Lacnospiraceae	Butyrate-producing bacteria, colonize the intestine mainly after weaning filling niches previously belonging to *Bifidobacteria* spp., but low amount of them also early in life may promotes the rapid change in the composition of the bacterial flora during weaning [82].	Reduced in microbiota in COVID-19 studies and negatively correlated with disease severity [65,67]. Negatively correlated with proteomic risk score for COVID-19 and cytokines [69].
Bacteroides	SCFAs producers from degradation of host glycans, mucine, oligosaccharides. Through their capsular polysaccharide they ✓ Activate CD4^+^T cells, ✓ Elicite appropriate cytokine production, ✓ Establish a proper T_H_1/T_H_2 balance, ✓ Promote adequate development of GALT. [68,83,84]. Through their membrane sphingolipids they ✓ Activate natural killer T (NKT) cells, ✓ Lead to rapid cytokine release, ✓ Facilitate bacterial clearance during infection. [85,86]. Bacteroides sphingolipids significantly decreased in the stool of IBD subjects and negatively correlated with gut inflammation. [86]. Role in counteract SARS-CoV-2? ✓ Stimulation of Treg cells and the release of anti-inflammatory cytokines [65,66,87], ✓ Decrease the expression of ACE receptors, impacting viral binding [65,66,87].	Negatively correlated with proteomic risk score for COVID-19 and with pro-inflammatory cytochines [65]. Negatively correlated with viral load [69].

* IL10 increase in the early phases of COVID-19 could represent a negative feedback mechanism to counteract other pro-inflammatory cytokines. On the contrary, when the disease evolves towards more severe clinical features, IL10 promotes inflammation and predicts a high mortality [88]. HMOs: human milk oligosaccharides. BF: breastfed. SCFAs: short chain fatty acids. ahRs: aryl hydrocarbon receptors. GALT: gut-associated lymphoid tissue. IBD: intestinal bowel diseases. INFα: interferon alpha. ACE: angiotensin-converting enzyme. IL: interleukin; T_H_1: T helper 1. T_H_2: T helper 2.

## Data Availability

Not applicable.
